# Family Support, Communication with Parents, and Adolescent Health Risk Behaviour: A Case of HBSC Study from Bulgaria and Lithuania

**DOI:** 10.3390/children12050654

**Published:** 2025-05-19

**Authors:** Elitsa Dimitrova, Apolinaras Zaborskis

**Affiliations:** 1 Department of Demography, Institute for Population and Human Studies, Bulgarian Academy of Sciences, 1000 Sofia, Bulgaria; 2 Plovdiv University Paisii Hilendarski, 4000 Plovdiv, Bulgaria; 3 Department of Preventive Medicine & Health Research Institute, Faculty of Public Health, Medical Academy, Lithuanian University of Health Sciences, A. Mickevičiaus 9, LT-44307 Kaunas, Lithuania; apolinaras.zaborskis@lsmu.lt

**Keywords:** family, parents, adolescents, health risk behaviour, (e)cigarette smoking, drunkenness, cannabis use, family support, communication with parents, structural equation modelling

## Abstract

*Objective:* This study aimed to explore the association between adolescents’ health risk behaviours (excessive use of alcohol, (e)cigarette smoking, cannabis use) and familial factors. A special objective of this study was to compare findings between Bulgarian and Lithuanian adolescents aged 15 years. *Material and Methods:* National samples from the WHO Health Behaviour in School-aged Children (HBSC) survey in 2021/2022 were analysed. The focus was on adolescents aged 15 (*n* = 64,349), including those from Bulgaria (*n* = 793) and Lithuania (*n* = 1137). The set of outcome variables included drunkenness, smoked cigarettes, e-cigarettes, and used cannabis (all variables were measured during the last 30 days); their indicators were child’s talking separately to their father and separately to their mother, as well as the four-item family support scale. All variables were dichotomised and their associations were analysed using Structural Equation Modelling with a WLSMV estimator. *Results:* In the total sample, the prevalence of drunkenness was 14.9%, cigarette smoking at 12.6%, e-cigarette smoking at 18.4%, and cannabis use at 5.9%; while in Bulgaria, in contrast to Lithuania, these behaviours were much more prevalent, at 27.0%, 29.9%, 29.8%, and 11.1%, respectively. The use of substances was significantly associated with selected familial factors, which were more pronounced among girls than boys in most subsamples. Low family support showed the strongest association with increased substance use (in the total sample, regression weight B varied from 0.231 to 0.382). Adolescents’ difficulty in talking to mother was more pronounced (B = 0.123 to 0.204) than difficulty in talking to their father (B = 0.058 to 0.140). Comparison of data samples from Bulgaria and Lithuania showed stronger relationships in Bulgarian adolescents compared to other countries, which are more pronounced among boys. In addition, among Bulgarian adolescents, easy communication with their father had an inverse association (increasing prevalence) with cannabis use. *Conclusions:* Adolescent health risk behaviours, such as use of substances, are associated with familial factors, including parent–teen communication and family support. Generally, these associations are more pronounced among girls than boys, and more evident among Bulgarian adolescents than their Lithuanian counterparts. Identifying environmental factors in families helps to plan interventions to prevent development of multiple health risk behaviours in adolescents.

## 1. Introduction

Adolescence is a critical developmental stage marked by significant physical, emotional, and psychological changes [1]. During this period, many young people engage in health risk behaviours such as alcohol use, smoking, and cannabis consumption. These behaviours can have long-term negative effects on their health and well-being. However, the presence of family support can play a crucial role in mitigating these risks. Research has consistently shown that adolescents who feel supported by their families—whether emotionally, socially, or through open communication—are less likely to engage in harmful behaviours [2].

Family support can take many forms, including emotional encouragement, advice, and practical assistance [3]. Studies indicate that adolescents who feel emotionally supported by their families are less likely to engage in behaviours like drinking [4], cigarette smoking [5,6], and drug use [7]. For instance, a supportive family provides a sense of security and self-worth that discourages adolescents from seeking validation through risky behaviours. Additionally, families that are actively involved in their children’s lives, offering guidance and setting clear boundaries, are more likely to foster an environment where harmful behaviours are less appealing.

A key factor in the association between family support and risk behaviour is communication [8]. Adolescents who can openly discuss their problems with their parents tend to make healthier decisions. Effective communication creates an atmosphere of trust, where teens feel safe enough to share their experiences, challenges, and concerns. In contrast, adolescents who feel that they cannot talk to their parents often turn to peer groups or external sources for support, which can sometimes encourage negative behaviour [9,10].

Parents, who show an interest in their children’s lives, such as discussing daily experiences, talking about emotions, or even making joint decisions, create a supportive atmosphere that reduces the temptation to engage in risky behaviours [11]. When parents are approachable and willing to listen without judgement, adolescents are more likely to seek their guidance when faced with challenges like peer pressure or stress. This can help prevent behaviours such as alcohol consumption, smoking, or drug use, which are often influenced by peer pressure or the desire to fit in.

Moreover, when families work together to set clear expectations and provide consistent reinforcement, adolescents are less likely to engage in risk behaviour. For example, a teenager who knows their parents are concerned about their well-being and are willing to discuss decisions openly may feel more confident in resisting peer pressure [12]. In such environments, teens are encouraged to make decisions that align with their values, even when faced with the temptation to follow the crowd.

The ease of talking with parents about problems, desires, and pressures is another vital aspect of reducing health risk behaviour. If adolescents feel comfortable discussing their challenges with their parents without fear of criticism or punishment, they are more likely to approach their family for advice and support when they encounter situations involving alcohol, smoking, or drugs [13]. In contrast, when adolescents feel that their parents are too strict, dismissive, or uninterested, they may struggle to communicate their concerns and may be more susceptible to risky behaviours in search of acceptance from peers. Families that build open, non-judgemental relationships foster an environment, where adolescents can make more informed, responsible choices. In these cases, adolescents are less likely to rely on unhealthy coping mechanisms such as substance use or smoking to deal with stress or emotional challenges.

Adolescence is a turning point in gender differences related to health behaviours. During this developmental stage, psychosomatic complaints increase in girls together with worsened emotional well-being, while health risk behaviours (e.g., injuries and accidents) become more prevalent among boys [14]. In terms of substance use, the recent trends outline a ‘catch-up’ pattern among girls engaged in substance use. Despite the diminishing gender differences in health risk behaviours, qualitative differences remain in terms of frequency and quantity in the use of harmful substances [14,15]. Gender plays also an important role in how adolescents respond to family support and how the ease of communication with parents affects young people’s behavioural health [13,16].

Most of the studies reviewed above focused on individual components of adolescent problem behaviour rather than examining multiple components simultaneously [17]. However, adolescents often engage in several risk behaviours at once, indicating that interventions should target multiple behaviours concurrently rather than in isolation. Several studies have explored patterns of multiple risk behaviours (MRBs) among adolescents—such as alcohol use, cigarette smoking, and drug use—in a more comprehensive manner. For instance, the Avon Longitudinal Study of Parents and Children [18] investigated how MRBs cluster in adolescents. The study found that MRBs tend to cluster by the number of behaviours rather than by forming distinct behavioural profiles. Similarly, the Korean Youth Risk Behaviour Survey [19], which analysed data from over 100,000 Korean adolescents, reported that 6.4% of males and 3.8% of females engaged in MRBs, including alcohol use, smoking, and illicit substance use. Increased age, low socioeconomic status, and mental health issues were associated with a higher likelihood of engaging in MRBs. These findings suggest that interventions should focus on the cumulative number of risk behaviours rather than targeting specific behaviour patterns. Studies such as those cited above and others [20] employed various Structural Equation Modelling (SEM) techniques to analyse the data. We considered SEM an appropriate method for examining the association between adolescent multiple health risk behaviour and familial factors. To our knowledge, no such study has yet been conducted to date.

Health Behaviour in School-aged Children (HBSC) is a cross-national study focused on adolescent health and well-being conducted in collaboration with the World Health Organisation (WHO) Regional Office for Europe [21,22]. At present, the HBSC study was implemented in more than 40 countries in Europe, Central Asia, and North America. The sample of each participating country includes adolescents aged 11, 13, and 15 years. The main topics covered by HBSC concern adolescents’ attitudes and experiences about mental health and well-being, physical activity, health risk behaviours, eating and dieting, electronic media communication, family culture, peer culture, leisure time, school experience, chronic conditions, etc. [21]. Communication with parents and family support are considered important factors of a social environment in the HBSC study [23].

Given the above provided background, the current study aimed to explore the association between multiple health risk behaviours (excessive use of alcohol, (e)cigarette smoking, cannabis use) taken comprehensively, that is, altogether, and several familial factors (ease of communication with parents and family support) in the total HBSC sample, as well as by comparing Bulgarian and Lithuanian adolescents aged 15 years, with a focus on gender differences. These two countries were chosen for the case study because they have not only many similarities, but also differences.

Bulgaria and Lithuania are Central and Eastern European countries that share many similarities in their past and recent societal development. In terms of trends in young people’s health behaviour, both countries show some differences related to lower prevalence of cigarette smoking, alcohol use, drunkenness, and cannabis use among Lithuanian adolescents compared to their Bulgarian counterparts [22]. In Lithuania, since the restoration of their independence in 1990, the concept of family has evolved alongside societal modernisation, legal reforms, and cultural shifts. Today, laws promote the active involvement of both parents in childcare (such as encouraging paternity leave), prohibit all forms of violence against children, and empower children to have a greater voice within the family. However, families continue to face challenges, including the growing difficulty of shielding children from the increasing availability of narcotic substances [24]. Similar changes in the concept of family were observed in Bulgaria related to the active promotion of positive parenting, a child-centred approach in policy making, as well as the adoption of the principle of the child’s best interest in legal documents and policies with the aim to promote child and family well-being [25].

## 2. Materials and Methods

### 2.1. Study Population

We used data from the eighth wave of HBSC surveys, conducted during the 2021/2022 school year across 44 regions (40 countries) in Europe, Central Asia, and Canada [21]. In all countries, an observational cross-sectional study design was applied. Research teams followed the standardised methodology outlined in the HBSC protocol [21]. The study collected data on health behaviours, health outcomes, and the social environments of adolescents aged 11, 13, and 15. Additional details about the HBSC study’s aims, theoretical framework, and survey methodology are available online [21] and in international reports [22,26].

The current study focused on the analysis of 15-year-old adolescents. A total of 64,349 records were selected, all of which contained complete and non-missing data used in the present analysis. Data were taken from 36 countries, including Bulgaria and Lithuania, with sample sizes of 793 and 1137 fifteen-year-olds, respectively. The data were obtained from the HBSC Data Management Centre at Bergen University, Norway. The HBSC protocol required ethical approval in each participating country. Since this study is a secondary analysis of existing datasets, with no direct participation from respondents, additional ethical approval was deemed unnecessary.

### 2.2. Variables

The outcome (dependent) variables measured substance use over the past 30 days. Alcohol use was assessed based on instances of ‘was drunken’ with the following response categories: 1 = never, 2 = once, 3 = 2–3 times, 4 = 4–10 times, and 5 = more than 10 times. Nicotine and cannabis use was evaluated through ‘cigarettes smoked’, ‘electronic (e-) cigarettes smoked’, and ‘cannabis used’, with the following response options: 1 = never, 2 = 1–2 days, 3 = 3–5 days, 4 = 6–9 days, 5 = 10–19 days, 6 = 20–29 days, and 7 = all days. In analyses, these variables were dichotomised into 0 = never and 1 = at least once.

The set of independent variables included only familial factors based on communication with parents and family support. Communication with parents was assessed using two questions. Adolescents were asked how easy it is for them to talk to their father and then to their mother about things that really bother them. Response options were as follows: 1 = very easy, 2 = easy, 3 = difficult, and 4 = very difficult. The present analysis included only those participants who reported talking to both biological parents. For each parent, the response categories ‘very easy’ and ‘easy’ were combined into a single category coded as 0 (easy), while ‘difficult’ and ‘very difficult’ were combined into a single category coded as 1 (difficult).

Family support was measured using the Multidimensional Scale of Perceived Social Support [27,28], adapted and validated for the HBSC study as the ‘Family Support Scale’. Adolescents responded to four statements: (1) ‘My family really tries to help me’, (2) ‘I get the emotional help and support I need from my family’, (3) ‘I can talk about my problems with my family’, and (4) ‘My family is willing to help me make decisions’. Response options ranged from 0 (‘very strongly disagree’) to 6 (‘very strongly agree’). Family support was assessed by summing the scores of responses to the statements, with higher total score indicating greater levels of family support. Based on the median value, a total score of 20 or higher was categorised as high perceived family support (=0), while a score below 20 was categorised as low perceived family support (=1). The models of associations were compared between adolescent gender and countries (Bulgaria and Lithuania).

### 2.3. Data Analysis

We conducted quantitative data analysis, utilising the whole sample (data combined from 36 countries, *n* = 64,349) as well as its subsamples from Bulgaria (*n* = 793) and Lithuania (*n* = 1137).

First, descriptive statistics were calculated to estimate the frequency (*n*), percentage (%), mean, standard deviation (SD), standard error (SE), and range of the variables. Bivariate analysis was performed to identify differences between respondent groups. The Chi-square test and z-tests were applied to compare percentages of categorical variables, while Odds Ratio (OR) was calculated to evaluate an association between the binary outcome variable and its predictor. A *p*-value ≤ 0.05 was considered statistically significant, and the confidence interval (CI) was set at 95%. These analyses were conducted using SPSS statistical software (version 21; IBM SPSS Inc., Chicago, IL, USA).

Then, Structural Equation Modelling (SEM) was applied to conduct analysis of relationship between adolescent use of substances and familial factors [29,30]. This approach served as an alternative to Multivariate Analysis of Variance (MANOVA) [31]. SEM offers several key advantages over MANOVA, particularly in complex modelling situations. For example, SEM can simultaneously estimate multiple relationships among various types of variables and evaluate how well the theoretical model fits the data. In contrast, MANOVA is limited to assessing mean differences across groups on multiple dependent continuous variables and does not provide model fit statistics.

SEM analysis was performed using MPLUS 7 (Muthén & Muthén, Los Angeles, CA, USA, 2012) [30]. Each behaviour was represented by a dichotomous variable; therefore, associations between variables were estimated using logistic regression and model parameters were estimated using the Weighted Least Squares Mean and Variance adjusted (WLSMV) estimator, which is specifically designed for categorical observed variables, including binary (e.g., yes/no) data. WLSMV is robust against violations of normality and provides adjusted standard errors and Chi-square test statistics, making it suitable for analysing non-continuous indicators.

The SEM model provided regression weights (B) showing the strength of the association between the connected variables. In this analysis, it was preferable to use the unstandardised regression weight B, as in logistic regression, it is directly related to the Odds Ratio (OR) through the equation: B = *ln* (OR). Squared multiple correlations (R^2^) were displayed for each endogenous variable, which is the proportion of variable variance that is accounted for by its indicators. SEM analysis with WLSMV estimator provides the following model fit indices to evaluate how well the model represents the data: Relative chi-square (χ2/df) and its *p*-value, Comparative Fit Index (CFI), Tucker–Lewis Index (TLI), and Root Mean Square Error of Approximation (RMSEA). The χ2/(degree of freedom df) statistic was used to assess the magnitude of the discrepancy between the sample and fitted covariance matrix, but this statistic is sensitive to sample size [32]. Its value of less than 3 or a non-significant *p*-value (*p* > 0.05) corresponds to an acceptable fit. The values of CFI and TLI close to 1 (≥0.90) and a RMSEA value below 0.08 show a good model fit to real data [32,33].

## 3. Results

### 3.1. Sample Characteristic

This study included a total of 64,349 adolescents from 36 countries. Their ages ranged from 14.6 to 16.5 years, with a mean age of 15.56 ± 0.37. The ratio of boys to girls was approximately equal across countries. Table 1 describes the characteristics of the study participants, classified by gender and country of interest (Bulgaria and Lithuania).

Across all countries included in the analysis, the percentage of fifteen-year-olds who experienced drunkenness at least once in the last 30 days did not differ significantly between boys and girls (Table 1). However, cigarette and e-cigarette use were 1–2 percentage points higher among girls, whereas cannabis use remained more common among boys.

Bulgarian and Lithuanian samples of fifteen-year-olds differed in the prevalence of drunkenness (27.0% vs. 14.9%, *p* < 0.001), cigarette smoking (29.9% vs. 17.3%, *p* < 0.001), and cannabis use (11.1% vs. 5.5%, *p* < 0.001), but did not differ in e-cigarette smoking (29.8% vs. 30.7%, *p* = 0.660). In both countries, gender differences were generally small. However, Bulgarian boys were more likely than girls to experience drunkenness (31.7% vs. 21.8%, *p* = 0.002) and use cannabis (14.5% vs. 7.4%, *p* = 0.001), while girls in this country have surpassed boys in smoking e-cigarettes (33.2% vs. 26.6%, *p* = 0.045).

Across all compared countries, it was more common for adolescents to find it very easy or easy to talk to their mother than to their father. This pattern was consistent across both sexes, though boys generally reported better communication with both parents than girls. A similar trend was observed among Bulgarian and Lithuanian adolescents. However, Bulgarian adolescents were more likely than their Lithuanian counterparts to report that they found it easy to talk to their father.

Regarding family support, significant gender differences were observed among fifteen-year-olds in 28 of 36 countries, with boys having a higher mean sum score. A significant gender difference was also found in Lithuanian adolescents but not in Bulgarian adolescents. However, the overall difference in family support between Bulgarian and Lithuanian adolescents, regardless of gender, was small (45.8% vs. 48.1%, *p* = 0.312).

### 3.2. Bivariate Associations

Table 2 presents the bivariate associations between adolescents’ health risk behaviours and family characteristics assessed using Odds Ratios (ORs). In the combined sample from all participating countries, all selected health risk behaviours were significantly associated with difficulty in talking to parents and with low family support. This pattern was evident in both boys and girls, but it was more pronounced among girls. Overall, the associations were stronger for difficulty in talking to mothers than difficulty in talking to fathers, and the association with low family support was even stronger than with difficulty in talking to either parent.

At the national level, across 36 countries, difficulty in talking to the father was significantly associated with drunkenness in 17 countries (excluding Bulgaria), cigarette smoking in 27 countries (excluding Bulgaria and Lithuania), e-cigarette smoking in 27 countries, and cannabis use in 17 countries (excluding Bulgaria and Lithuania). Difficulty in talking to the mother had significant associations in even more countries, with drunkenness in 25 countries, cigarette smoking in 31 countries (excluding Lithuania), e-cigarette smoking in 29 countries, and cannabis use in 24 countries (excluding Lithuania). Low family support was a significantly associated with alcohol, cigarette, e-cigarette, and cannabis use in the majority of countries—specifically in 32, 35, 33, and 32 countries. However, in Bulgaria, according to the bivariate analysis, low family support had no significant relationship with e-cigarette smoking.

In the total sample, as previously noted, the examined associations were stronger among girls than boys. A similar pattern was observed in the Lithuanian data. However, as shown in Table 2, the pattern differed markedly in the Bulgarian sample. In this sample, most of the significant associations were found only among boys.

### 3.3. Multivariate Analysis with SEM Model

**Figure 1:**
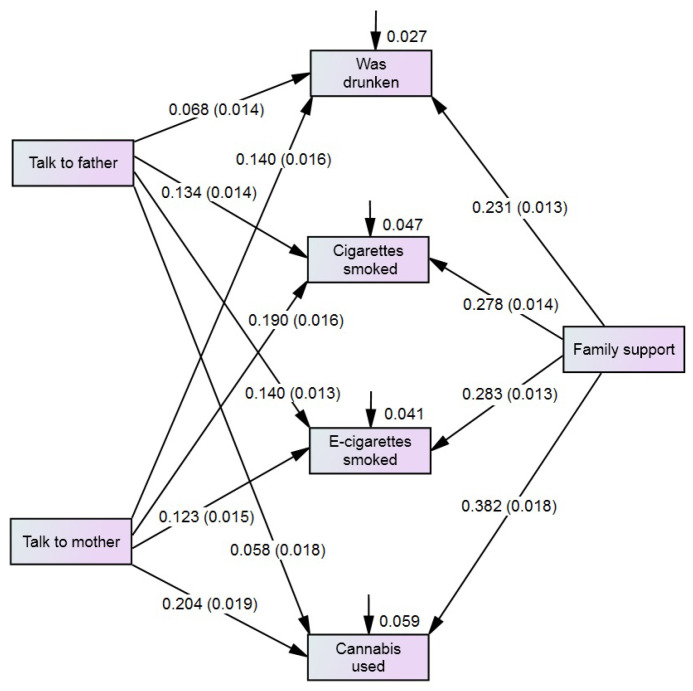
SEM model to analyse the association between family factors and adolescent health risk behaviour. Total sample (*n* = 64,349)—Health Behaviour in School-aged Children (HBSC) 2021/2022. Notes: The numbers on pathways are regression weights and standard errors; the numbers next to the boxes are squared multiple correlations; correlations between variables are not shown.

To clarify the mechanisms underlying relationships between adolescent multiple health risk behaviour and familial predictors, we used a Structural Equation Modelling (SEM) analysis. A hypothetical model illustrating the relationships between study variables is presented in Figure 1. Characteristics of this model were estimated for the entire sample (*n* = 64,349). Their statistical evaluation is presented in Table 3. The model included all possible relationships (paths) among variables as saturated model; therefore, fit statistics were not needed, as the model perfectly reproduced the observed covariance matrix.

The presented model confirmed the findings obtained from the analysis of bivariate associations (ORs) in the total sample (see Table 2). First, the consumption of all substances was significantly associated with familial factors. Second, low family support showed the strongest association with increased substance use (B = 0.231 to 0.382). Third, adolescents’ difficulty in talking to their parents had a weaker effect on substance use compared to family support, wherein difficulty in talking to the mother was more pronounced (B = 0.123 to 0.204) than difficulty in talking to the father (β = 0.058 to 0.140).

### 3.4. Multi-Group Analysis of Relationships

In this study, the feature of SEM multi-group analysis allowed the examination of differences in model structure between boys and girls, as well as between countries. Results of this analysis are shown in Table 3.

The test to check the equality of regression weights between groups revealed a significant overall difference between adolescents from Bulgaria and Lithuania (*p* < 0.001), as well as between boys and girls in the entire sample (*p* < 0.001) and the sample of Bulgaria (*p* = 0.007). The structural weights of the model were invariable when comparing boys and girls in the sample of Lithuania (*p* = 0.563). The goodness-of-fit statistics of SEM model in multi-group analyses perfectly met all the criteria for all comparisons, except the Chi-square test due to large sample size.

This analysis revealed several gender differences, particularly in communication with the father. In the total sample, girls who reported difficulty in talking to their father were more likely to engage in substance use than boys, whereas the opposite pattern was observed regarding communication with the mother. Additionally, girls who perceived low family support reported health risk behaviours more frequently than boys. Among Lithuanian adolescents, these patterns were weak. In contrast, among Bulgarian adolescents, health risk behaviours in boys appeared to be more strongly associated with family risk factors than in girls. In Bulgaria, communication with the mother was especially important for boys: difficulty in talking to their mother was strongly linked to an increased risk of substance use. Interestingly, Bulgarian adolescents who reported no difficulty talking to their father were more likely to use cannabis compared to their peers who reported having difficulty in talking to their father. In the case of SEM analysis, this difference was significant (B = −0.457, SE = 0.146, *p* = 0.002), while binary analysis showed only a trend for this difference (OR = 0.89, 95% CI: 0.54; 1.48, *p* = 0.656).

## 4. Discussion

The present study focussed on adolescents’ health risk behaviours that included excessive use of alcohol, smoking of cigarettes and e-cigarettes, and cannabis use. The main objective was to assess their association with several familial factors, such as easy communication with parents and family support. The study also aimed to identify differences in associations between boys and girls, and to examine these associations between Bulgarian and Lithuanian adolescents as a case study.

The novelty of our study lies in the modern SEM data analysis approach, which is able to analyse multiple relationships simultaneously [29,30]. For control, we also evaluated these relationships using bivariate analysis, calculating ORs. The same relationship trends were found, but with different significance levels. Since the logistic regression estimator was used in the SEM analysis, the differences between the two calculation methods can be tested using the formula B = *ln* (OR). In the multivariate SEM analysis, for the Bulgarian and Lithuanian samples, which were smaller in size, the strength of some relationships was weaker and less reliable than in the bivariate analysis. This is consistent with the multivariate analysis [31].

The results from the analysis revealed significant relationships between selected health risk behaviours and familial factors in the total sample. Overall, health risk behaviours among girls were more strongly associated with difficulties in communicating with parents and with low family support than among boys. However, case studies from Bulgaria and Lithuania revealed exceptions to this pattern, highlighting country-specific variations. These results are in line with existing studies, revealing gender patterns in adolescent health risk behaviours, i.e., girls having healthier behaviours (in excessive alcohol consumption and cannabis use) [34] and boys being more engaged in addictive risk behaviours [35]. Various familial factors such as family activities and parental behaviours [36], as well as family connectedness and parental involvement and interaction with children, were identified as “protective assets for [adolescent] health” [37].

The case study reveals that the prevalence of drunkenness, cigarette smoking, and cannabis use was higher among Bulgarian adolescents than among peers in Lithuania, except for e-cigarette smoking. Gender differences in adolescent substance use were generally small in both countries. The associations between familial and health risk behaviours were weak among Lithuanian boys and girls, while among Bulgarian boys, difficulty in communication with their mother was significantly associated with higher risk of drunkenness, (e)cigarette smoking, and cannabis use. These results align with existing studies, showing that the relationship between parental communication and adolescent substance use varied by gender and substance [16]. The results also show an opposite relationship between communication with father and cannabis use among Bulgarian boys. In particular, Bulgarian boys who reported easy communication with their fathers were more likely to use cannabis. This could be related to perceptions of less authoritarian and disciplining parenting style being tied to the ease of communication with the father. Societal norms and cultural expectations shape the gendered nature of the interaction between parents and children as part of the intergenerational transmission of gender role models. Existing studies reveal that fathers more often use authoritative parenting style for girls, featured by warmth and nurturing relationships, and more authoritarian parenting style for sons, featured by stronger rule enforcement and discipline [38,39]. Findings from country-focused studies also demonstrated similar features of family culture and the intergenerational gender role transmission for boys and girls [40,41].

In the total sample, low family support showed the strongest association with increased substance use among adolescents. There were also significant gender differences in the association between family support and adolescents’ substance use. In particular, high family support was associated with a decrease in the risk of drunkenness, cigarette smoking, and cannabis use among Bulgarian boys and a decreased risk of e-cigarette smoking and cannabis use among Lithuanian girls. These findings show that the supportive relationships in the family are particularly important for young people in reducing the risk of engagement in substance use and demonstrate the distinctive effect of family cohesion and family interaction for adolescent health behaviours [42,43]. Future research could explore what specific aspects of family support (e.g., emotional versus decision-making support) are more relevant with regard to prevention and intervention programmes, which also take into account the bounding of adolescent health risk behaviours [44].

### Strengths and Limitations

The present study was strengthened using a large and culturally diverse sample of adolescents. The uniform methodology of the HBSC study, however, ensured the comparability of data between countries. The findings of the study increased our knowledge in adolescents’ health risk behaviour in the context of family functioning and contributed to the existing literature in this field. In this study, we intended to give a sense of importance to SEM analysis in the understanding of the system of relationships between familial factors and a set of adolescent behaviours. SEM offers several advantages over other methods of multivariate analysis (e.g., MANOVA), particularly when dealing with complex relationships between variables. The key advantages of this statistical analysis approach include simultaneous testing of multiple relationships, reducing measurement error; separating measurement error from true scores, which leads to more reliable estimates; providing a range of model fit indices to evaluate how well the model represents the data [29,31]. Additionally, SEM allows for the inclusion of latent (unobserved) variables by using multiple observed indicators. However, we did not use this advantage for the unobserved variable named “family support” that was built on four-item scale. Instead, we assessed only the dichotomised summed score of this variable. This solution was chosen to have a simpler model and an easier interpretation of the results.

We also hereby acknowledge other limitations of the present study. The HBSC study has a cross-sectional design, and this allows us to explore only associations between the studied variables, but not causation. It is conceivable, for example, that adolescents who do not engage in risk behaviours are viewed more positively by their parents, who in turn find it easier to talk with and support their teens. The HBSC study also relies only on adolescents’ self-reported data and is thus a subject to potential response bias. For example, using sensitive questions, such as questions about substance use, can be affected by the possibility for social fear bias in adolescent responses. Every effort was made to minimise that possibility by ensuring strict anonymity of respondents. The HBSC study does not include a measure about the parenting styles, which may influence the ease of communication and the interactions between parents and children, thereby affecting young people’s engagement in substance use as well. Meanwhile, other variables from the HBSC study, such as family socioeconomic status, were excluded from the model to avoid complicating its analysis and interpretation. Another limitation of the study is that the ease of communication with parents is measured by a single question asked separately for the mother and father. Finally, our analysis included only those adolescents who had both parents. This did not allow us to explore in detail the different aspects of communication and interaction between parents and children in different family structures.

## 5. Conclusions

The present study revealed significant relationships between adolescent health risk behaviours, such as drunkenness, cigarette and e-cigarette smoking, and cannabis use, and familial factors, including parent–teen communication and family support. Structural Equation Modelling demonstrated that these associations differ in their properties and strength when examined within a multi-risk behaviour framework compared to analyses based on individual risk factors. This approach enabled a deeper understanding of the gendered nature of these relationships. Overall, health risk behaviours among girls were more strongly associated with difficulties in communicating with parents and with low family support than among boys. However, case studies from Bulgaria and Lithuania revealed exceptions to this pattern, highlighting country-specific variations.

## Figures and Tables

**Table 1:** Occurrence of health risk behaviours during the last 30 days and rate of positive family characteristics in total sample and subsamples (%)—Health Behaviour in School-aged Children (HBSC) 2021/2022.

Characteristics	Groups of Respondents								
	Total Sample			Selected Countries		Bulgaria		Lithuania	
	Boys and Girls (*n* = 64,349)	Boys (*n* = 31,070)	Girls (*n* = 33,279)	Bulgaria (*n* = 793)	Lithuania (*n* = 1137)	Boys (*n* = 413)	Girls (*n* = 380)	Boys (*n* = 592)	Girls (*n* = 545)
Was drunken	14.9	14.9	14.9	27.0	14.9 ***	31.7	21.8 **	14.0	15.8
Cigarette smoked	12.6	12.1	13.1 ***	29.9	17.8 ***	29.1	30.8	18.4	16.1
E-cigarette smoked	18.4	17.3	19.3 ***	29.8	30.7	26.6	33.2 *	30.4	31.0
Cannabis used	5.9	6.8	5.1 ***	11.1	5.5 ***	14.5	7.4 ***	6.6	4.2
Easy talk to father	63.6	73.1	54.7 ***	73.0	59.9 ***	79.2	66.3 ***	72.0	46.8 ***
Easy talk to mother	78.1	82.0	74.5 ***	79.3	75.9	79.7	78.8	80.9	70.5 ***
High family support	53.1	57.4	49.0 ***	45.8	48.1	44.3	47.4	52.2	43.7 **

Notes: Significance of the difference between groups of respondents: * *p* < 0.05, ** *p* < 0.01, *** *p* < 0.001.

**Table 2:** Association between health risk behaviours and family characteristic: Odds Ratio (95% confidence interval), in the total sample and subsamples—Health Behaviour in School-aged Children (HBSC) 2021/2022.

Risky Behaviour by Family Characteristic	Groups of Respondents								
	Total Sample			Selected Countries		Bulgaria		Lithuania	
	Boys and Girls (*n* = 64,349)	Boys (*n* = 31,070)	Girls (*n* = 33,279)	Bulgaria (*n* = 793)	Lithuania (*n* = 1137)	Boys (*n* = 413)	Girls (*n* = 380)	Boys (*n* = 592)	Girls (*n* = 545)
Was drunken on:									
Talk to father (difficult vs. easy)	1.46 *** (1.40; 1.53)	1.35 *** (1.26; 1.45)	1.60 *** (1.51; 1.71)	1.15 (0.81; 1.62)	1.63 *** (1.17; 2.26)	1.20 (0.73; 1.98)	1.31 (0.79; 2.18)	1.65 * (1.02; 2.69)	1.60 * (1.00; 2.56)
Talk to mother (difficult vs. easy)	1.65 *** (1.52; 1.73)	1.61 *** (1.50; 1.74)	1.69 *** (1.58; 1.80)	2.34 *** (1.63; 3.36)	1.49 * (1.04; 2.14)	2.70 *** (1.65; 4.41)	2.05 ** (1.19; 3.56)	1.31 (0.75; 2.29)	1.61 * (1.00; 2.60)
Family support (low vs. high)	1.74 *** (1.67; 1.82)	1.61 *** (1.51; 1.71)	1.90 *** (1.78; 2.02)	1.99 *** (1.44; 2.76)	1.46 * (1.04; 2.03)	2.59 *** (1.66; 4.04)	1.39 (0.85; 2.28)	1.21 (0.76; 1.92)	1.75 * (1.07; 2.84)
Cigarette smoked on:									
Talk to father (difficult vs. easy)	1.78 *** (1.70; 1.86)	1.62 *** (1.51; 1.74)	1.93 *** (1.81; 2.06)	1.35 (0.97; 1.89)	1.08 (0.79:1.47)	1.32 (0.79; 2.19)	1.36 (0.86; 2.14)	0.97 (0.61; 1.54)	1.33 (0.84; 2.11)
Talk to mother (difficult vs. easy)	2.00 *** (1.90; 2.10)	1.95 *** (1.80; 2.11)	2.02 *** (1.89; 2.16)	2.51 *** (1.76; 3.58)	1.40 (0.99; 1.97)	3.22 *** (1.96; 5.29)	1.93 * (1.16; 3.21)	1.17 (0.70; 1.95)	1.74 * (1.08; 2.79)
Family support (low vs. easy)	2.09 *** (1.99; 2.19)	1.88 *** (1.76; 2.02)	2.29 *** (2.14; 2.45)	1.98 *** (1.45; 2.72)	1.44 * (1.06; 1.97)	2.77 *** (1.74; 4.39)	1.45 (0.93; 2.25)	1.19 (0.79; 1.81)	1.94 * (1.19; 3.16)
E-cigarette smoked on:									
Talk to father (difficult vs. easy)	1.67 *** (1.61; 1.74)	1.52 *** (1.43; 1.62)	1.77 *** (1.68; 1.87)	1.49 * (1.06;2.07)	1.41 * (1.09; 1.82)	1.34 (0.80; 2.26)	1.48 (0.95; 2.32)	1.19 (0.81; 1.75))	1.69 ** (1.17; 2.45)
Talk to mother (difficult vs. easy)	1.74 *** (1.66; 1.82)	1.66 *** (1.54; 1.76)	1.77 *** (1.67; 1.88)	1.70 ** (1.19; 2.44)	1.48 ** (1.11; 1.97)	2.76 *** (1.67; 4.57)	1.03 (0.61; 1.74)	1.39 (0.91; 2.14)	1.56 * (1.06; 2.30)
Family support (low vs. high)	1.94 *** (1.86; 2.01)	1.70 *** (1.60; 1.80)	2.17 *** (2.05; 2.94)	1.16 (0.85; 1.57)	1.54 ** (1.19; 1.99)	1.94 ** (1.23; 3.06)	0.74 (0.48; 1.14)	1.29 (0.91; 1.83)	1.89 ** (1.30; 2.77)
Cannabis used on:									
Talk to father (difficult vs. easy)	1.78 *** (1.67; 1.90)	1.75 *** (1.59; 1.92)	2.23 *** (2.01; 2.47)	0.89 (0.54; 1.48)	1.64 (0.98; 2.74)	1.19 (0.62; 2.28)	0.77 (0.33; 1.81)	1.48 (0.75; 2.92)	3.31 *** (1.21;9.05)
Talk to mother (difficult vs. easy)	2.25 *** (2.10; 2.41)	2.19 *** (1.98; 2.41)	2.54 *** (2.30; 2.80)	3.91 *** (2.46; 6.20)	1.66 (0.97; 2.87)	4.64 *** (2.59; 8.31)	3.13 *** (1.42; 6.93)	1.51 (0.71; 3.19)	2.27 (0.98; 5.27)
Family support (low vs. high)	2.75 (2.56; 2.96)	2.60 *** (2.37; 2.85)	3.26 *** (2.91; 3.66)	3.46 *** (2.04; 5.87)	2.57 *** (1.46; 4.56)	4.81 *** (2.36; 9.78)	1.99 (0.88; 4.53)	1.81 (0.93; 3.54)	8.66 *** (2.01; 37.3)

Notes: Statistical significance of the deviation from 1.00: * *p* < 0.05, ** *p* < 0.01, *** *p* < 0.001.

**Table 3:** Several estimates from SEM multi-group analysis—Health Behaviour in School-aged Children (HBSC) 2021/2022.

Characteristics	Groups of Respondents								
	Total Sample		Selected Countries			Bulgaria		Lithuania	
	Boys and Girls (*n* = 64,349)	Boys (*n* = 31,070)	Girls (*n* = 33,279)	Bulgaria (*n* = 793)	Lithuania (*n* = 1137)	Boys (*n* = 413)	Girls (*n* = 380)	Boys (*n* = 592)	Girls (*n* = 545)
Path	Regression weights B (standard errors)								
Was drunken on:									
Talk to father	0.068 (0.014) ***	**0.021 (0.022)**	0.119 (0.019) ***	−0.149 (0.114)	0.196 (0.107)	−0.136 (0.129)	0.005 (0.165)	0.281 (0.162)	0.111 (0.154)
Talk to mother	0.140 (0.016) ***	0.170 (0.024) ***	0.119 (0.021) ***	0.456 (0.125) ***	0.083 (0118)	0.434 (0.134) ***	0.351 (0.185)	−0.010 (0.192)	0.136 (0.153)
Family support	0.231 (0.013) ***	0.208 (0.019) ***	0.257 (0.019) ***	0.312 (0.104) **	0.095 (0.105)	0.548 (0.117) ***	**0.080 (0.154)**	0.032 (0.139)	0.190 (0.162)
Cigarettes smoked on:									
Talk to father	0.134 (0.014) ***	0.080 (0.022) ***	0.180 (0.018) ***	−0.047 (0.112)	−0.101 (0.118)	−0.237 (0.130)	0.034 (0.159)	−0.085 (0.154)	−0.064 (0.162)
Talk to mother	0.190 (0.016) ***	0.217 (0.025) ***	0.171 (0.021) ***	0.419 (0.125) ***	0.151 (0.118)	0.524 (0.137) ***	0.334 (0.185)	0.085 (0.174)	0.204 (0.162)
Family support	0.278 (0.014) ***	0.252 (0.020) ***	0.305 (0.020) ***	0.289 (0.103) **	0.193 (0.106)	0.472 (0.121) ***	**0.080 (0.154)**	0.112 (0.135)	0.321 (0.171)
E-cigarettes smoked on:									
Talk to father	0.140 (0.013) ***	0.098 (0.021) ***	0.163 (0.018) ***	0.157 (0.109)	0.087 (0.091)	**−0.130 (0.131)**	0.317 (0.154) *	0.015 (0.136)	0.166 (0.131)
Talk to mother	0.123 (0.015) ***	0.140 (0.028) ***	0.106 (0.020) ***	0.272 (0.124)	0.110 (103)	0.550 (0.148) ***	**−0.038 (0.184)**	0.145 (0.154)	0.081 (0.141)
Family support	0.283 (0.013)	0.229 (0.018) ***	0.335 (0.018) ***	−0.021 (0.103)	0.189 (0.089) *	0.145 (0.119)	−0.308 (0152)	0.117 (0.119)	0.279 (0.137) *
Cannabis used on:									
Talk to father	0.058 (0.018) ***	0.051 (0.026) *	0.154 (0.026) ***	−0.457 (0.146) **	0.052 (0.147)	−0.397 (0.155) **	−0.481 (0.261)	0.092 (0.183)	0.200 (0.327)
Talk to mother	0.204 (0.019) ***	0.210 (0.028) ***	0.211 (0.027) ***	0.733 (0.152) ***	**0.059 (0.150)**	0.750 (0.148) ***	0.608 (0.317)	0.004 (0.201)	0.040 (0.244)
Family support	0.382 (0.018) ***	0.386 (0.024) ***	0.392 (0.028) ***	0.485 (0.145) **	0.392 (0.147) **	0.483 (0.151) ***	0.081 (0.277)	0.273 (0.172)	0.782 (0.369) *
Variable	Squared multiple correlations (R^2^)								
Was drunken	0.027	0.020	0.036	0.064	0.020	0.112	0.019	0.017	0.028
Cigarettes smoked	0.047	0.035	0.059	0.067	0.014	0.104	0.026	0.004	0.039
E-cigarettes smoked	0.041	0.027	0.054	0.021	0.021	0.062	0.032	0.010	0.043
Cannabis used	0.059	0.055	0.079	0.145	0.046	0.145	0.069	0.027	0.170
		Test to check the equality of regression weights between groups							
Degree of freedom		12		12		12		12	
Chi-squared		164.76		53.826		21.448		12.411	
*p*-value		<0.001		<0.001		0.015		0.563	
		Goodness-of-fit characteristics							
Chi-squared/degree of freedom		16.021; *p* < 0.001		6.002; *p* < 0.001		2.462; *p* = 0.042		1.136; *p* = 0.480	
TLI		0.986		0.911		0.905		0.981	
CFI		0.974		0.942		0.912		0.975	
RMSEA (90% CI)		0.027 (0.018; 0.036)		0.052 (0.041; 0.070)		0.048 (0.024; 0.072)		0.021 (0.009; 0.032)	

Notes: Significance of the difference from the zero value: * *p* < 0.05, ** *p* < 0.01, *** *p* < 0.001; Underlined and bolded estimates differ significantly (*p* < 0.05) between the respondents’ groups.

## Data Availability

The data presented in this study are available upon reasonable request from the HBSC Data Management Centre, University of Bergen, Norway (dmc@hbsc.org).
